# Foreign Bodies in Trachea and Bronchial Tubes

**Published:** 1913-07

**Authors:** F. G. Hodgson

**Affiliations:** Atlanta, Ga.


### Journal-Record of Medicine

Successor to Atlanta Medical and Surgical Journal, Established 1855
and Southern Medical Record, Established 1870

**OWNED BY THE ATLANTA MEDICAL JOURNAL COMPANY**

PUBLISHED MONTHLY

*Official Organ Fulton County Medical Society, State Examining Board, Presbyterian Hospital, Atlanta, Birmingham and Atlantic Railroad Surgeons' Association, Chattahoochee Valley Medical and Surgical Association, Etc.*

EDGAR BALLENGER, M. D., Editor
BERNARD WOLFF, M. D., Supervising Editor
A. W. STIRLING, M. D., C. M., D. P. H.; J. S. HURT, B. Ph., M.D.
GEO. M. NILES, M. D., W. J. LOVE, M. D., (Ala.); Associate Editors
E. W. ALLEN, Business Manager

**COLLABORATORS**

DR. W. F. WESTMORLAND, General Surgery
F. W. McRAE, M. D., Abdominal Surgery
H. F. HARRIS, M. D., Pathology and Bacteriology
E. B. BLOCK, M. D., Diseases of the Nervous System
MICHAEL HOKE, M. D., Orthopedic Surgery
CYRUS W. STRICKLER, M. D., Legal Medicine and Medical Legislation
E. C. DAVIS, A. B., M. D., Obstetrics
E. G. JONES, A. B., M. D., Gynecology
R. T. DORSEY, JR., B. S., M. D., Medicine
L. M. GAINES, A. B., M. D., Internal Medicine
GEO. C. MIZELL, M. D., Diseases of the Stomach and Intestines
L. B. CLARKE, M. D., Pediatrics
EDGAR PAULIN, M. D., Opsonic Medicine
THEODORE TOEPEL, M. D., Mechano Therapy
R. R. DALY, M. D., Medical Society
A. W. STIRLING, M. D., Etc., Diseases of the Eye, Ear, Nose and Throat
BERNARD WOLFF, M. D., Diseases of the Skin
E. G. BALLENGER, M. D., Diseases of the Genito-Urinary Organs

VOLUME LX JULY, 1913 No. 4

### FOREIGN BODIES IN TRACHEA AND BRONCHIAL TUBES.

BY F. G. HODGSON, M. D., ATLANTA, GA.

The question of foreign bodies in the air passages is a very old one, and the literature on this subject is very extensive. I shall not go into a detailed discussion of this subject, as it can be found in any of the modern text books on Surgery. I simply wish to report four cases successfully operated upon by me, and to show the specimens.

CASE 1.—Baby Harris, only 10 months old. Two days ago she pulled an old brass pin, 1½ inches long, out of a doll, put the pin into her mouth and aspirated it into her trachea. This was followed by a violent attack of coughing and dyspnoea. This subsided, but was followed by a cough, and some bloody mucus was spit up, and she had occasional attacks of choking. The baby was taken to one doctor of good diagnostic ability, but as the baby was showing no symptoms at the time, he decided that the parents were unnecessarily alarmed, and told them that there was nothing the matter. Not being satisfied, they took the baby to Dr. John Hurt, who, after getting a careful history, decided to take an x-ray photograph, and this located the pin. He then referred the case to me. Physical signs in the chest were negative. I did a low tracheotomy under general anesthesia, and was able to grasp the pin with forceps and remove it. I made an attempt to close the wound in trachea, but was unable to do so completely and a general emphysema of the skin on neck and chest began to develop, so the wound was opened again and left open. It rapidly granulated and was closed at the end of a week. The baby made a rapid and uneventful recovery.

CASE 2.—Lena H., age 5 years. Aspirated a grain of dry corn into the trachea. This was followed by choking, coughing and dyspnoea. These attacks were repeated at frequent intervals. The corn evidently being freely movable in the trachea. There was a muco-purulent expectoration. Physical signs in the chest were negative, but the history was so typical that a tracheotomy was done under general anesthesia. Upon opening the trachea, the child coughed up a considerable quantity of thick muco-pus. The corn could not be located with various forceps, so a tracheotomy tube was put in to keep the trachea open. This was removed in 12 hours, and the wound left wide open, covered with gauze. The child was acutely ill for 24 hours. High temperature, rapid pulse and very rapid respirations, but in 36 hours the grain of corn was coughed out into the gauze covering the tracheotomy wound, and from then on her convalescence was rapid. The wound healing by granulation.

CASE 3.—Geneva M., age 2 years, 36 hours previously aspirated a grain of dry corn. Was taken to a physician who tried emetics, then chloroform,—inverted the child trying to shake out the corn. An attempt was also made to use a bronchoscope, but this also was unsuccessful. Case referred to me by Dr. Phinizy Calhoun. Physical signs showed harsh sterterous breath sounds over both lungs, evidently due to free foreign body in trachea. Chloroform anesthesia. Low tracheotomy—instruments inserted into trachea, caused violent coughing, and grain of corn came into wound and was removed by forceps. Wound left open. Convalescence uneventful.

CASE 4.—Baby W., age 21 months, was given some green peanuts to play with. Naturally, she put them in her mouth, and in a few minutes her father noticed she was blue in the face and, choking violently. She was inverted and beaten upon the back, and coughed up some of the peanuts.

She was better, but continued to have attacks of coughing, choking and dyspnoea. She was seen by a doctor, who said nothing could be done, but gave her some medicine to prevent inflammation of the lungs from setting in. Every time the baby coughed, she was violently jagged up and down and beaten upon the back to prevent choking to death. For five days the parents and friends had kept that poor child going night and day, and beating it almost constantly. When I saw her, the mother would not leave her still long enough for me to examine the baby's chest. The poor child was almost exhaused, by the frequent attacks of choking, and the violent treatment she was receiving. A broncho-pneumonia had developed at base of right lung. Temp. 103, pulse 120, resp. 36. It was a poor risk, but it seemed the child would die if not relieved. Under light general anesthesia, low tracheotomy was done—forceps inserted and a peanut grasped, which was mashed, and a piece of the covering of the nut removed. The wound was left open, covered only by a moist piece of gauze. The child was desperately ill for 18 hours. Temp. 105, pulse 160, resp.-60. She was extremely restless and hard to keep in bed. Had it not been for the constant and excellent care of a good trained nurse, I feel sure the child would have died. After 24 hours, however, she began to convalesce, and was soon out of danger. There are several practical points I wish to call attention to, viz:

A.—The fact that foreign bodies in the air passages are often coughed up spontaneously, does not justify undue delay in operating. The dangers of waiting are greater than the dangers of operating. The smallest foreign body (the peanut), in this series, caused the greatest distress, caused a broncho-pneumonia, and the child nearly died, due to the complications of delay.

B.—No attempt should be made to close the tracheotomy wound. A tracheotomy tube is unnecessary, and may prevent the expulsion of the foreign body, if it has not been removed. A silk traction suture can be introduced into each side of the wound in trachea, and used to keep it open.

C.—Recent advances made in the use of the bronchoscope, make it a very valuable aid in locating and removing foreign bodies in the trachea and bronchial tubes.

